# The Repeating, Modular Architecture of the HtrA Proteases

**DOI:** 10.3390/biom12060793

**Published:** 2022-06-07

**Authors:** Matthew Merski, Sandra Macedo-Ribeiro, Rafal M. Wieczorek, Maria W. Górna

**Affiliations:** 1 Structural Biology Group, Biological and Chemical Research Centre, Faculty of Chemistry, University of Warsaw, Żwirki i Wigury 101, 02-089 Warsaw, Poland; 2 Instituto de Investigação e Inovação em Saúde and Instituto de Biologia Molecular e Celular (IBMC), Universidade do Porto, 4200-135 Porto, Portugal; sribeiro@ibmc.up.pt; 3 Faculty of Chemistry, University of Warsaw, Pasteura 1, 02-093 Warsaw, Poland; wieczorek@chem.uw.edu.pl

**Keywords:** HtrA protease, protein repeat, PA clan, serine protease, protein evolution

## Abstract

A conserved, 26-residue sequence [AA(X_2_)[A/G][G/L](X_2_)GDV[I/L](X_2_)[V/L]NGE(X_1_)V(X_6_)] and corresponding structure repeating module were identified within the HtrA protease family using a non-redundant set (N = 20) of publicly available structures. While the repeats themselves were far from sequence perfect, they had notable conservation to a statistically significant level. Three or more repetitions were identified within each protein despite being statistically expected to randomly occur only once per 1031 residues. This sequence repeat was associated with a six stranded antiparallel β-barrel module, two of which are present in the core of the structures of the PA clan of serine proteases, while a modified version of this module could be identified in the PDZ-like domains. Automated structural alignment methods had difficulties in superimposing these β-barrels, but the use of a target human HtrA2 structure showed that these modules had an average RMSD across the set of structures of less than 2 Å (mean and median). Our findings support Dayhoff’s hypothesis that complex proteins arose through duplication of simpler peptide motifs and domains.

## 1. Introduction

**Figure 1:**
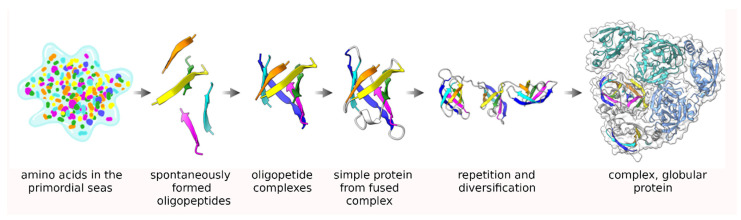
An illustration of Dayhoff’s hypothesis about the origin of proteins [9,17]. From left to right, starting from individual, spontaneously formed amino acids in the Archaean seas, short oligopeptides formed spontaneously which then organized into homogenous complexes and eventually fused into a single transcript module, probably after being encoded in the genome. Duplication and repetition of these modules along with drift in their sequence and function eventually gave rise to complex, globular proteins.

Generally, the arrangement of amino acids in proteins is seemingly random (complex), although exceptions exist where notable patterns can be discerned in the amino acid sequence, such as low-complexity proteins [1] or protein repeats [2,3,4]. Proteins also usually adopt distinct three-dimensional structures and a wide variety of these have been reported in the public repository of the Protein Data Bank (PDB) [5]. A combination of elements (sequence, secondary structure, fold, and three-dimensional structure) comprise the architecture of a protein. However, the three-dimensional structure itself tends to be the most conserved aspect of the protein as both the sequence and function of the protein evolve much more quickly [6], although it has been suggested that the folds themselves are fossils [7] of the early, Archean proteins which may have evolved before the appearance of the last universal common ancestor (LUCA) [8]. Five decades ago, based on the earliest protein structures, it was hypothesized that these primitive peptides formed oligomeric groups in solution which eventually fused into single peptide transcripts to give rise to the early, modern proteins which then over time gradually diverged into more complex forms [9,10,11,12,13]. This process of oligomerization followed by fusion has also been suggested to have given rise to repeat proteins, which are composed of a set of repeating structures and sequences of 20–60 amino acids in length, which may have, over time, evolved into complex, globular proteins (Figure 1) [2,14,15]. While there are a number of well-known repeat protein types, they have generally received less researcher attention than globular proteins that have more complex structural architectures despite estimates suggesting that about a quarter of all known proteins have at least some repeat protein character [16]. This raises the obvious question as to what is obscuring the presence of all these expected protein repeats in structural databases such as the PDB, especially given the possibility that the early, ancestral proteins were all at least repeat-like [17,18,19].

Protein families that are widely distributed across the three kingdoms of life are likely to have roots deep in evolutionary time [8,15], possibly even as far back as the Archean, pre-LUCA period, and may be, in essence, representatives of such preserved fossil architectures. One such protein family could be the HtrA family of proteases. The high temperature requirement A (HtrA) proteases are stress response, housekeeping proteases widely distributed throughout nature [20]. Notable examples of this family include DegP [21] and DegS [22] in prokaryotes, Deg1 in plants [23], and HtrA2 in humans [24,25,26,27]. Structurally, HtrA proteases are members of the PA clan of serine proteases (including such notable examples as chymotrypsin A and thrombin) which contain a pair of six-stranded β-barrels [28]. HtrA proteases additionally have one or more C-terminal PDZ-like domains [25,29], an 80–100 amino acid long protein interaction domain found in many different protein families [30]. In prokaryotes, DegP [21] forms large 12- and 24-mer complexes while DegS [22] exists as a simple trimer. In humans, the chromosomally encoded HtrA2 protease, linked to Parkinson’s disease [24], functions as a housekeeping protease within the mitochondria [26]. Damage to the mitochondrial membranes results in leakage of HtrA2 into the cytoplasm, where it digests peptide inhibitors of apoptosis leading to cell death [31]. HtrA2 has been shown to have an unusually high melting temperature [26] and to preferably cleave unfolded substrate ensembles [32]. HtrA2 is maintained in a resting closed state and its activation mechanism is a set of sequential steps that are initiated by the binding of a hydrophobic motif to the PDZ-like domain, followed by exposure of the substrate binding site on the protease domain and activation of the proteolytic activity.

**Figure 2:**
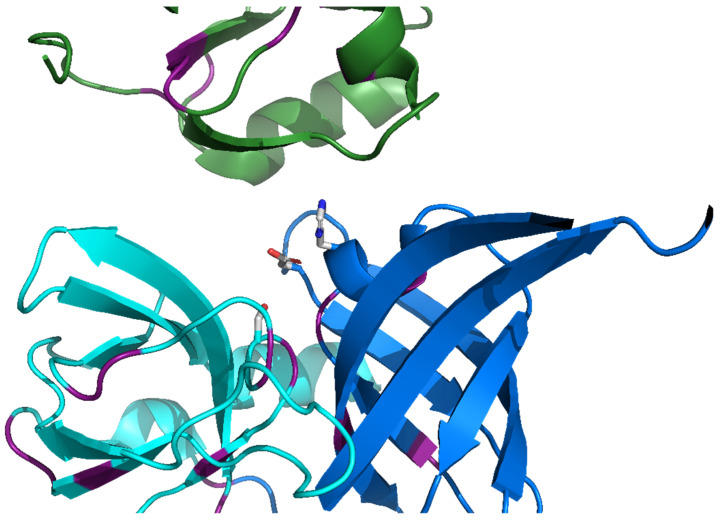
The active site in the HtrA proteases is separate between the modules. Cartoon diagram of human HtrA2 (PDB ID 5m3n [26]) showing the N-terminal protease (blue), C-terminal protease (cyan) and PDZ-like (green) modules. The catalytic triad of His198, Asp 228, and Ser306 are shown as sticks with light grey carbon, blue nitrogen, and red oxygen atoms. Those residues which correspond to conserved canonical repeat residues are indicated in purple (Figure S1).

We have recently reported [33] a survey of all the known protein sequences using a self-homology detection method based on DOTTER [34]. This allowed us to identify a number of protein families which had a notable amount of self-similarity, including the HtrA protease family. More detailed examination confirmed the initial detection of the repeating amino acid sequence and we were able to correlate the sequence repeats with a six-strand antiparallel β-barrel structure that occurred at least three times in the monomeric structure of the protease (twice in the protease domain, a feature of the PA clan of serine proteases [28]) (Figure 2) and once in each PDZ-like domain. These results suggest that the PDZ-like domain evolved from repetition of this basic barrel structure in the PA clan serine proteases.

## 2. Materials and Methods

As previously reported, all known proteins (UniRef90 [35]) were examined for self-homology using a modified version of DOTTER [33,34]. This analysis found a number of proteins with significant self-homology that were in a protease Do-like cluster. HtrA proteases were then collected from the PDB [5]. Short sequences (less than 200 residues) were removed and the remainder were filtered at the 90% sequence identity threshold with CD-HIT [36], leaving 20 unique structures (i.e., 2zle, 2z9i, 3gdv, 3nzi, 3pv5, 3qo6, 4a9g, 4fln, 4ic5, 4ic6, 4ri0, 4ynn, 5fht, 5ilb, 5jyk, 5t69, 5zvj, 6jjo, 6z05, 7co3). The locations of probable sequence repeats were identified by reverse calculation of the DOTTER plots of these protein sequences where each residue was assigned its maximal self-homology score. The high scoring regions from all the proteins were separated and the frequency of each amino acid at each position was calculated; those positions which had strong biases towards a single, or a pair of similar, amino acids were noted. This process identified a 26-residue repeating sequence. Multiple sequence alignment of these repeats with MUSCLE [37] (Figure S1) helped to clarify the repeated sequence in which 13 of the 26 positions were conserved, specifically [AA(X_2_)[A/G][G/L](X_2_)GDV[I/L](X_2_)[V/L]NGE(X_1_)V(X_6_)], which can also be represented as AA--[A/G][G/L]--GDV[I/L]--[V/L]NGE-V------. This repeat was then used for further sequence searches.

The statistical significance of this sequence repeat was verified by comparison of the repeat pattern to a randomly generated sequence. A score estimate for the random sequence was defined by a binomial (Bernoulli) model. The random chance for success at each position was defined as the probability for the expected amino acid at that position for each of the 13 defined positions. More explicitly, suppose a set of coin flips (Bernoulli trials) such that each event will have 13 trials and each of those trials have a probability of success defined by the natural frequency of the amino acids that are acceptable in that position. The score for each event is defined by the number of successes that occur in the event. For each success, a score of 1/P(x) where P(x) is the probability of finding an acceptable amino acid in that position is given while failures get a score of 1. For positions with 2 acceptable amino acids, the probability is the sum of the two amino acid frequencies. This allowed a success score to be defined for any actual sequence as

Score = Π(T)

where the score value for success T(S) in any position is

T(S) = 1⁄P(x)

and for failure T(F) is

T(F) = 1

Failures are given a score of one in order to not modify the score (i.e., a sequence with no matches to the defined repeat sequence received a score of 1). To further verify this model, a 9996060-residue length of random amino acid sequence was generated by the Sequence Manipulation Suite [38] and each 26-residue sequence unit was compared to the repeat sequence to generate an estimate of the probability for the repeat sequence to appear randomly (Figures S2 and S3). Sequences with a score greater than 90,000 appeared with a frequency of less than 1 per 1000 residues, in agreement with the theoretical binomial model.

The repeat sequence was then identified in a multiple sequence alignment (MUSCLE [37]) in the set of protein structures (Figure S1). These were comprised of a pair of six-stranded barrels in the protease domain and a partial, four-stranded barrel in each PDZ-like domain (Figure S4). The PDB structures were divided into the individual barrel structures and compared by structural alignment in PyMol [39]. The sequence repeats did not share a common secondary structure. However, a shared common antiparallel β-barrel structure could be identified within the structures, which was associated with the sequence repeats (Figure S5). While generally, the alignment of the domains to each other was poor using PyMol, the three domains from a specific human HtrA2 protease (PDB ID 5m3n) could all be superimposed when aligned in PyMol. The N terminal protease, C terminal protease, and PDZ domain modules from all the other example HtrA proteases could then be aligned to the equivalent module from the 5m3n structure and a good superimposition was achieved (Figure S6). The modules were also compared by TM-align [40].

## 3. Results

**Figure 3:**
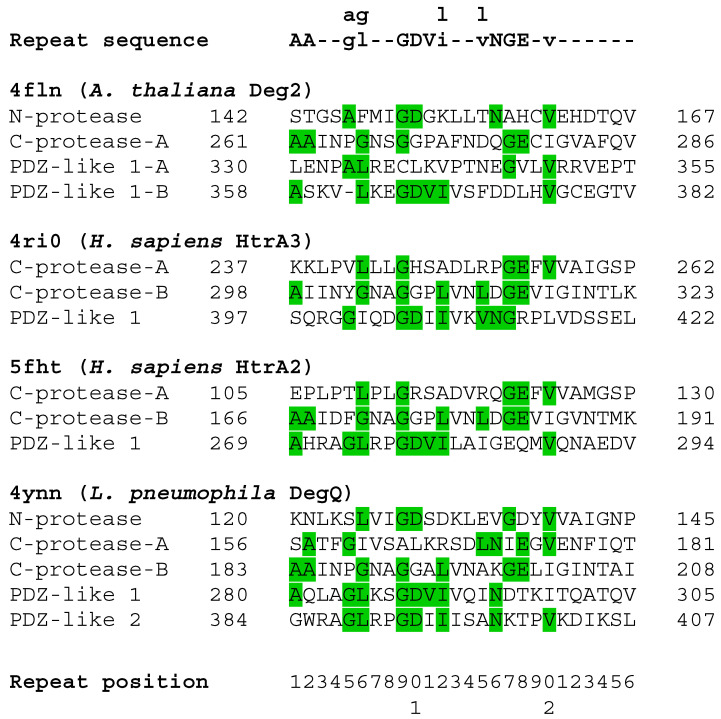
Identification of the sequence repeats in the HtrA proteases. The canonical sequence [AA(X_2_)[A/G][G/L](X_2_)GDV[I/L](X_2_)[V/L]NGE(X_1_)V(X_6_)] is shown on top in bold, with an additional residue shown for the four positions which have two possible canonical residues. Residues that match the canonical sequence are highlighted in green. The PDB ID, species, and protein name are given along with the module in which the sequence is located. When a module has two copies of the sequence repeat, the most N-terminal is denoted as A and the other as B. The beginning sequence position (using the PDB numbering) is to the left of the sequence while the ending position is given to the right of the sequence.

**Figure 4:**
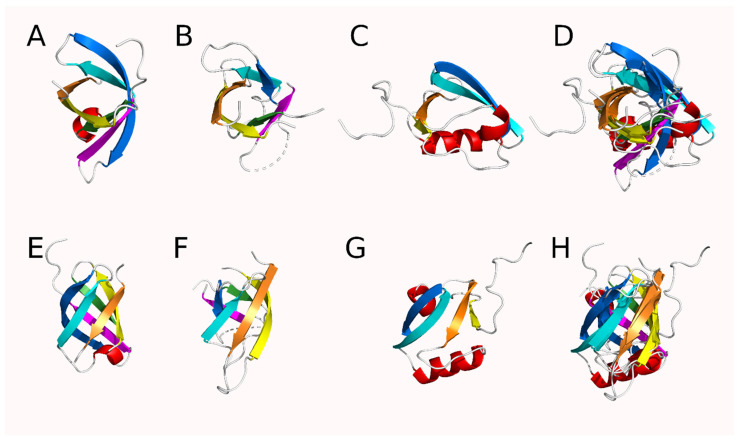
Cartoon diagram of the modules from HhoA, an HtrA protease from *Synechocystis* sp. PCC 6803 (PDB ID 5t69) showing the conserved structures of the HtrA modules (RMSD to PDB ID 7co3: mean = 1.748 Å, median = 1.816 Å). Strands are colored orange, yellow, green, blue, and magenta in order from N to C in the protease modules and in the equivalent spatial position in the PDZ-like module. Helices are colored red and coil regions are white. Top-down views of the (**A**) N-terminal protease module, (**B**) C-terminal protease module, (**C**) PDZ-like module and (**D**) all three modules superimposed. Side views of the (**E**) N-terminal protease module, (**F**) C-terminal protease module, (**G**) PDZ-like module, and (**H**) all three modules superimposed.

Using a modified form of DOTTER to analyze protein self-homology, the presence of notable self-homology was detected in the HtrA proteases. Reverse calculation of the homology plots produced by DOTTER [34] using HtrA protease sequences from the PDB allowed the detection of a putative 26 amino acid repeat [AA(X_2_)[A/G][G/L](X_2_)GDV[I/L](X_2_)[V/L]NGE(X_1_)V(X_6_)] and alignment of the repeating regions with MUSCLE [37] produced an initial estimate of the repeating sequence with 13 of the 26 positions being definable (Figure 3 and Figure S1). Comparison to a random sequence and theoretical comparison to a Bernoulli model for cumulative probability (Figures S2 and S3) suggested that high scoring matches (typically five or more matching positions) should be relatively uncommon, occurring randomly only once every 1031 residues, which is much less than once per protein as the average HtrA protease monomer is 350–450 residues long [41]. Examination of the sequence unique HtrA proteases (90% ID) clearly identified three or more of these repeats in each monomer, corresponding roughly to two in the protease domain and one in each PDZ-like domain (Figure S4) (some HtrA proteases such as DegP have two copies of the PDZ-like domain [30]). The protease and PDZ-like domains within the HtrA proteases did not have significant self-homology (mean = 18.2%, median = 15.3% for protease and PDZ-like 1 domains, N = 19 proteins (Table S1)). Only one protein, *Legionella pneumophila* DegQ (PDB ID 4ynn) [42], had greater than 30% identity between its protease and PDZ-like domains (34.0% ID). Low shared sequence identity has been previously noted in PDZ domains [43]. Further visual inspection of the structures identified a common alternating antiparallel six-strand β-barrel module in the structures (Figure 4 and Figure S5). There is a shift in register in the PDZ-like module in which the first beta strand occurs outside of the barrel structure and the barrel remains unclosed as it contains only four additional strands (Figure S5). The sixth strand is also rotated out of the structure or deleted, depending on the species. The alternating anti-parallel pattern present in the protease modules is maintained in the PDZ-like module but formally reversed as the designation of positive and negative strands is arbitrary. RMSD comparison of these isolated structures using PyMol found a poor structural similarity among the β-barrels with a mean RMSD of 4.1 Å between the two protease domains and mean RMSD values of 8.8 Å and 9.2 Å between the N-terminal protease module or the C-terminal protease module and the first PDZ-like domain, respectively (Table S2). The superposition of the PDZ-like and protease modules was poor but their small size makes the RMSD values appear better than they are. Analysis by TM-align [40] suggested good agreement between the protease domains (TM scores = 0.552 (mean), 0.549 (median) for the protease modules and 0.652 (mean), 0.638 (median) for the PDZ-like modules; RMSD = 2.80 Å (mean), 2.78 Å (median) for the protease domains and 2.28 Å (mean), 2.22 Å (median) for the PDZ-like domains). However, there was poor correspondence when the protease modules were compared to the PDZ-like modules or vice-versa (TM-score = 0.298 (mean), 0.299 (median); RMSD = 3.72 Å (mean), 3.70 Å (median)) (Table S3). The modules from one human HtrA2 structure (PDB ID 5m3n [26]) could be structurally aligned after manual examination (Figure 4, Table S3). The aligned modules from this specific PDB structure could then be used as “targets” for the corresponding modules in the other proteins. When this was done, the structural differences were generally minimized and good structural alignments could be achieved (mean = 2.9 Å, 2.9 Å, 1.9 Å; median = 2.5 Å, 2.8 Å, 1.7 Å for the N-terminal protease, C-terminal protease, and PDZ-like domain modules, respectively) (Table S3).

## 4. Discussion

Fundamentally, the HtrA proteases are repeat proteins. A 26-residue sequence repeat associated with an anti-parallel β-barrel structure is clearly identifiable in the HtrA proteases (structures shown in Figure 2 and Figure 4). While the repeating sequences are far from perfect [44], the frequency of matches to the defined canonical sequence is statistically significant (Figures S2 and S3). The individual modules can be structurally aligned to a set of target modules to a good average RMSD (<3 Å) between the β-barrel structural modules within a given protein (Figure 4, Table S3). Repetitions of both sequence and structure in combination with the presence of two copies of a β-barrel in the PA clan of serine proteases [28] (to which HtrA proteases belong) and all three modules having a peptide binding function strongly suggests that the HtrA proteases are the result of a set of repetitions of the ancestral β-barrel module followed by mutation and functional change in the third (by sequence order) module present in the PDZ-like domain(s) [45].

The evolution of the modern HtrA protease structure from an ancestral β-barrel precursor, possibly an Archean, pre-LUCA protease [8], via the PA clan ancestor [28] offers an elegant solution to the problem of the origin of structural complexity of this family of proteases from a simple ancestor as suggested by Dayhoff’s hypothesis [9,17]. It is currently unknown if the ancestral module itself was an active protease or if it simply had a peptide binding function and developed into an active protease after the duplication at the origin of the PA clan as the catalytic triad of the HtrA proteases is spread across the two protease modules. For example, in human HtrA2 [25], *E. coli* DegP [46], and *A. thaliana* Deg2 [47], the catalytic serine is found in the C-terminal protease module, while the other two members of the triad are present in the first module. The PDZ-like module is likely derived from one of these modules as it contains several divergent structural features compared to the protease modules. The protease modules have six strands comprising its ꞵ-barrel, while only four of these are present in the PDZ-like domain module [45] along with an additional N-terminal strand which is rotated out of the barrel structure (Figure S5). There are also many PA clan proteases which lack the PDZ-like domain, suggesting that it evolved later. Therefore, while it is not undisputable, it seems likely that the PDZ-like module is a product of the duplication of one of the protease modules rather than the protease being derived from the PDZ-like domain. This may be an incorrect assumption, however, given the amount of lateral gene transfer that occurs in prokaryotes [48,49].

By analyzing the conserved self-homology patterns in the HtrA proteases, we were able to identify the simple, repeating β-barrel architecture present in this family. To the best of our knowledge, this repeating architecture has gone unremarked upon despite the fact that structures of these proteins have been publicly available for 20 years [25,29] and the widely recognized pair of β-barrels present in the PA clan of proteases. This was likely at least partially due to the general difficulty in identifying protein repeats [50,51,52]. In this specific case, there are one (or two) sequence repeats present in each of the β-barrel structural modules (Figure S1), and a small but notable discrepancy between the sequence and structural repetitions, which would contribute to the difficulty in identifying these repeats. However, a discrepancy between sequence and structural repeats is not uncommon in repeat proteins [53]. Additionally, automated structural alignment methods had difficulty in detecting the similarity between the modules, even after the repeats had been unambiguously identified. The low sequence similarity between the members of the family or the different repeat modules as well as the low sequence conservation within the repeats themselves likely contributed to this detection issue, as did the variability in the assignment of the secondary structures in the protein structure models themselves (Tables S2 and S3). It is also worth noting that several standard structural alignments failed to properly superimpose the protease modules with PDZ-like modules., However, they could be convinced to superimpose the modules when a properly superimposed “target” structure was used (Figure S6, Table S3). We must also note that even the method used here has its limitations. The length is defined by a relatively well-conserved valine at position 26 (Figure 3). However, this valine does not occur as frequently in the data set as the defined canonical residues, and certainly not at the 40% frequency used to define other canonical repeat residues [54], but its presence did help to define the length of the HtrA repeats described here.

Nevertheless, the successful detection of this overlooked repeat architecture in a well-studied family of proteins using self-homology does not imply that this method for repeat detection cannot be further improved. While the method was able to find the sequence repeats fairly easily, they did not correspond to the structural repeat and identification of that required significant human intervention. Even when the β-barrel was recognized, the movement of the first and last strand out of the barrel and the spatial rearrangement of the strands prevented accurate matching of the β-barrels using structural alignment algorithms without human optimization. Clearly, improvements in the automation of the structure search strategies would be beneficial here, since simple removal of the coil regions did not improve detection ability, quite likely due to discrepancies in identification of secondary structural features in the crystallographic models (Table S3). Finally, despite identification of these repeating modules, it still cannot be indisputably determined which module is the most ancestral and which are derived.

Despite these caveats, the conserved, repeating architecture of the HtrA proteases is clearly identifiable in the family. Self-homology analysis was able to identify this architecture which had gone overlooked for decades, a clear success for this method of repeat detection. This repeat architecture shows an elegant method to generate complex protein structures from simple oligopeptide building blocks and might serve to inform protein engineering efforts. This repeat detection methodology can (and will) be applied to other well-studied protein families and potentially identify their underlying repeat architectures.

## Data Availability

Protein structures analyzed in this work are publicly available in the PDB (www.rcsb.org, accessed 1 April 2022).

## Supplementary Materials

The following supporting information can be downloaded at: https://www.mdpi.com/article/10.3390/biom12060793/s1, Figure S1: MUSCLE 3.8 Alignment of HtrA proteases, Figure S2: Frequency of HtrA repeats in random protein sequences, Figure S3: Expected protein length of a random sequence matching that score to occur, Figure S4: Identification of the β–barrel structures in the HtrA proteases, Figure S5: Secondary structures of the HtrA modules, Figure S6: Structures of the HtrA modules, Table S1: Domain sequence similarity comparison, Table S2: Unmodified RMSD comparison table, Table S3: β-barrel comparison summary table.
