# Supplementary material for: The Repeating, Modular Architecture of the HtrA Proteases
Source: Biomolecules. 2022 Jun 7;12(6):793. doi: 10.3390/biom12060793 (PMC9221053; doi:10.3390/biom12060793)
Supplement: Supplementary file 1 [file biomolecules-12-00793-s001.zip › biomolecules-1762636-SI update-6.8.pdf]

Supplimental Material for: “The Repeating, Modular Architecture of the HtrA Proteases”

Authors: Matthew Merski[1*], Sandra Macedo-Ribeiro[2], Rafal Wieczorek[3], Maria W. Górna[1]

Affilitations:

1 - Structural Biology Group, Biological and Chemical Research Centre, Faculty of Chemistry, University of Warsaw, Żwirki i Wigury 101, 02-089 Warsaw, Poland

2 - Instituto de Investigação e Inovação em Saúde and Instituto de Biologia Molecular e Celular (IBMC), Universidade do Porto, Porto, Portugal

3 - Faculty of Chemistry, University of Warsaw, Pasteura 1, 02-093 Warsaw, Poland

* - corresponding author, email: merski@gmail.com (+48 225 526 642) (M.M.); mgorna@chem.uw.edu.pl (M.W.G.)

<u>Figure S1:</u> MUSCLE 3.8 Alignment of HtrA Proteases

Multiple sequence alignment of unique HtrA sequences from the PDB. The β-barrel structures are identified with light grey highlighting (Figure S4). Potential sequence repeat regions are identified by dark grey highlighting while residues matching the cannonical repeat sequence are shown with green highlighting. Structure 4a9g lacked explicit identification of its secondary structures within the PDB model and PDB ID 3nzi lacked a modeled PDZ-like domain so those β-barrel modules were not indicated with grey highlighting.

```
4fln            --------------------------NAESSNPPQKMAFKAFG-----------------
5ilb            --------------------------GHDASF----------------------------
5jyk            -------------MGSSHH-----HHHHSSGLVPRGSHMASMTGGQQMGRGSEFELLNNE
5fht            ------------------------------MAVP--------------------------
3nzi            -------------MG---------------------------------------------
4ri0            ------------------------------------------------------------
6z05            ------------------------------------------------------------
2z9i            -----------------------------ANMPP--------------------------
5zvj            ------------------------MVDAFTTSKVT-------------------------
3gdv            -------------MRGSHH-----HHHHGRSLNPLS------------------------
7co3            MPKALRFLGWPVLVGVLLALLIIQHNPELVGLPRQEVHVEQ-------------------
3pv5            -------------MRGSHH-----HHHHGSAEPPN-------------------------
4ynn            -------------MRGSHH-----HHHHGSAQDLT-------------------------
4a9g            -----------------------SIPGQVADQAP--------------------------
2zle            ------------------------------------------------------------
6jjo            -------------MGSSHH-----HHHHSSGLVPRGSHMA--------------------
5t69            ---------------------GIDPFTMADDLPPAPVI----------------------
4ic5            -------------MGSSHH-----HHHHSSGLVPRGSHMASAL-----------------
4ic6            -------------MGLGDP-----SVATVEDVSPT-------------------------
3qo6            -------------------------MAAFVVSTPK-------------------------

4fln            -----------SPKKEKKESLSDFSRDQQTDPAKIHDASFLNAVVKVYC-----------
5ilb            ----------------------------------------LNAVVKVYC-----------
5jyk            SEAGNQRTSSPERSRSRLHHSDTKNGDCSNGMIVSTTTESIPAAPSWETVVKVVPSMDAV
5fht            --------------SPPPASPRSQYNFIADVVEKT-----APAVVYIEI-----------
3nzi            --------------QEDPNSLRHKYNFIADVVEKI-----APAVVHIEL-----------
4ri0            --------------LHQLSSPRYKFNFIADVVEKI-----APAVVHIEL-----------
6z05            --------------VLSYHDS----------IKDA-----KKSVVNISTSKTITRANRPS
2z9i            -------------------------GSVEQVAAKV-----VPSVVMLET-----------
5zvj            --------------LSTTGNAQEPAGRFTKVAAAV-----ADSVVTIES-----------
3gdv            --------------TPQFDSTDETPASYNLAVRRA-----APAVVNVYN-----------
7co3            --------------APLLSRLQEGPVSYANAVSRA-----APAVANLYTTKMVSKPSHPL
3pv5            --------------MPSMAPV----------LKNI-----MPAIVNVAVQGYLP------
4ynn            -----------------------NMPSLAPVLKNA-----MPAIVNVAVQGYLPNNM---
4a9g            --------------LPSLAPM----------LEKV-----LPAVVSVRVEGTASQGQK--
2zle            -------------AETSSATTAQQMPSLAPMLEKV-----MPSVVSINVEGSTTVNTPR-
6jjo            --------------ETSSATTAQQMPSLAPMLEKV-----MPSVVSINVEGSTTVNTPR-
5t69            --------------TAQASVPLTSESFVAAAVSRS-----GPAVVRIDTETVVTRRTDPI
4ic5            -----------EQFKEKEEELEEEEERNVNLFQKT-----SPSVVYIEA-----------
4ic6            --------------VFPAGPLFPTEGRIVQLFEKN-----TYSVVNIFD-----------
3qo6            --------------KLQTDEL-----ATVRLFQEN-----TPSVVYITN-----------
                                                          :
```

```
4fln    ----------THTAPDYSLPWQKQ----------------------RQFTSTGSAFMIG-
5ilb    ----------THTAPDYSLPWQKQ----------------------RQFTSTGSAFMIG-
5jyk    V-----KVFCVHTEPNFSLPWQRK----------------------RQYSSGSSGFIIG-
5fht    --------LDRHPFLGREVPI-----------------------------SNGSGFVVAA
3nzi    --------FRKLPFSKREVPV-----------------------------ASGSGFIVSE
4ri0    --------FLRHPLFGRNVPL-----------------------------SSGSGFIMSE
6z05    PLDDFFNDPYFKQFFDFDFPQRKGKNDK------------------EVVSSLGSGVIISK
2z9i    -------------DLGRQSEE-------------------------------GSGIILSA
5zvj    --------------VSDQEGM------------------------------QGSGVIVDG
3gdv    --------RGLNTNSHNQLEIR----------------------------TLGSGVIMDQ
7co3    F-----DDPMFRRFFGDNLPQQK-----------------------RMESSLGSAVIMSA
3pv5    ------NDVTPPGSAGNDEENQPNNRPPQSRMPEKG----------RKFESIGSGVIIDP
4ynn    ------ASGNADDDDGENSKQPSRIPEKG-----------------RKFESIGSGVIIDP
4a9g    ------IPEEFKKFFGDDLPDQPA----------------------QPFEGLGSGVIINA
2zle    ------MPRNFQQFFGDDSPFCQEGSPFQSSPFCQGGQGGNGGGQQQKFMALGSGVIIDA
6jjo    ------MPRNFQQFFGDDSPFCQEGSPFQSSPFCQGGQGGNGGGQQQKFMALGSGVIIDA
5t69    L-----DDPFFQEFFGRSFPVPPRE---------------------RRIAGQGSGFIIDN
4ic5    ------IEL-PKTSSGDILTDEEN----------------------GKIEGTGSGFVWDK
4ic6    ------VTLRPQLKMTGVVEIP---------------------------EGNGSGVVWDG
3qo6    ------LAVRQDAFTLDVLEVP---------------------------QGSGSGFVWDK
                                                            .*..:

4fln    -DGKLLTNAHCVEHD--T---------QVKVKRRGDDR-KYVAKVLVRGVDCDIALLSVE
5ilb    -DGKLLTNAHCVEHD--T---------QVKVKRRGDDR-KYVAKVLVRGVDCDIALLSVE
5jyk    -GRRVLTNAHSVEHH--T---------QVKLKKRGSDT-KYLATVLAIGTECDIALLTVT
5fht    -DGLIVTNAHVVADR--R---------RVRVRLLSG-D-TYEAVVTAVDPKADIATLRIQ
3nzi    -DGLIVTNAHVVTNK--H---------RVKVELKNG-A-TYEAKIKDVDEKADIALIKID
4ri0    -AGLIITNAHVVSSN--SAAPGRQ---QLKVQLQNG-D-SYEATIKDIDKKSDIATIKIH
6z05    -DGYIVTNNHVVDDA--D---------TITVNLPGSDI-EYKAKLIGKDPKTDLAVIKIE
2z9i    -EGLILTNNHVIAAA--AKPPLGSPPPKTTVTFSDG-R-TAPFTVVGADPTSDIAVVRVQ
5zvj    -RGYIVTNNHVISEA--ANNPSQF---KTTVVFNDG-K-EVPANLVGRDPKTDLAVLKVD
3gdv    -RGYIITNKHVINDA--D---------QIIVALQDG-R-VFEALLVGSDSLTDLAVLKIN
7co3    -EGYLLTNNHVTAGA--D---------QIIVALRDG-R-ETIAQLVGSDPETDLAVLKID
3pv5    NNGVIITNDHVIRNA--S---------LITVTLQDG-R-RLKARLIGGDSETDLAVLKID
4ynn    KNGIIITNDHVIRNA--N---------LITVTLQDG-R-RLKARLIGGDSETDLAVLKID
4a9g    SKGYVLTNNHVINQA--Q---------KISIQLNDG-R-EFDAKLIGSDDQSDIALLQIQ
2zle    DKGYVVTNNHVVDNA--T---------VIKVQLSDG-R-KFDAKMVGKDPRSDIALIQIQ
6jjo    DKGYVVTNNHVVDNA--T---------VIKVQLSDG-R-KFDAKMVGKDPRSDIALIQIQ
5t69    -SGIILTNAHVVDGA--S---------KVVVTLRDG-R-TFDGQVRGTDEVTDLAVVKIE
4ic5    -LGHIVTNYHVIAKL--ATDQFGLQRCKVSLVDAKGTRFSKEGKIVGLDPDNDLAVLKIE
4ic6    -QGYIVTNYHVIGNALSRNPSPGDVVGRVNILASDGVQKNFEGKLVGADRAKDLAVLKVD
3qo6    -QGHIVTNYHVIRGA--S---------DLRVTLADQ-T-TFDAKVVGFDQDKDVAVLRID
            ::** *                    :             :   .   *:* : :
```

```
4fln    SEDFWKGAEPLRLGHLPRLQDSVTVVGYPLGG----DTISVTKGVVSRIEVT-----SYA
5ilb    SEDFWKGAEPLRLGHLPRLQDSVTVVGYPLGG----DTISVTKGVVSRIEVT-----SYA
5jyk    DDEFWEGVSPVEFGDLPALQDAVTVVGYPIGG----DTISVTSGVVSRMEIL-----SYV
5fht    TKEP-LPTLPLGRSADVRQGEFVVAMGSPFAL-----QNTITSGIVSSAQRPAR---DLG
3nzi    HQGK-LPVLLLGRSSELRPGEFVVAIGSPFSL-----QNTVTTGIVSTTQRGGK---ELG
4ri0    PKKK-LPVLLLGHSADLRPGEFVVAIGSPFAL-----QNTVTTGIVSTAQREGR---ELG
6z05    ANN--LSAITFTNSDDLMEGDVVFALGNPFGV-----GFSVTSGIISALNK------DNI
2z9i    GVSG-LTPISLGSSSDLRVGQPVLAIGSPLGL-----EGTVTTGIVSALNRPVSTTGEAG
5zvj    NVDN-LTVARLGDSSKVRVGDEVLAVGAPLGL-----RSTVTQGIVSALHRPVPLSGEGS
3gdv    ATGG-LPTIPINARRVPHIGDVVLAIGNPYNL-----GQTITQGIISATGR------IGL
7co3    LKN--LPAMTLGRSDGIRTGDVCLAIGNPFGV-----GQTVTMGIISATGR------NQL
3pv5    AKN--LKSLVIGDSDKLEVGDFVVAIGNPFGLNSFGNSQSATFGIVSALKR------SDL
4ynn    AKN--LKSLVIGDSDKLEVGDYVVAIGNPFGLNSFGNSQSATFGIVSALKR------SDL
4a9g    NPSK-LTQIAIADSDKLRVGDFAVAVGNPFGL-----GQTATSGIVSALGR------SGL
2zle    NPKN-LTAIKMADSDALRVGDYTVAIGNPFGL-----GETVTSGIVSALGR------SGL
6jjo    NPKN-LTAIKMADSDALRVGDYTVAIGNPFGL-----GETVTSGIVSALGR------SGL
5t69    PQGSALPVAPLGTSSNLQVGDWAIAVGNPVGL-----DNTVTLGIISTLGRSAA---QAG
4ic5    TEGRELNPVVLGTSNDLRVGQSCFAIGNPYGY-----ENTLTIGVVSGLGREI----PSP
4ic6    APETLLKPIKVGQSNSLKVGQQCLAIGNPFGF-----DHTLTVGVISGLNRDI----FSQ
3qo6    APKNKLRPIPVGVSADLLVGQKVFAIGNPFGL-----DHTLTTGVISGLRREIS---SAA
                  .         :   .:* *          : * *::*

4fln    HGSSDLLGIQIDAAINPGNSGGPAFNDQGECIGVAFQV--YRSEETE------NIGYVIP
5ilb    HGSSDLLGIQIDAAINPGNSGGPAFNDQGECIGVAFQV--YRSEETE------NIGYVIP
5jyk    HGSTELLGLQIDAAINSGNSGGPAFNDKGKCVGIAFQS--LKHEDAE------NIGYVIP
5fht    LPQTNVEYIQTDAAIDFGNAGGPLVNLDGEVIGVNTMK--VTA----------GISFAIP
3nzi    LRNSDMDYIQTDAIINYGNSGGPLVNLDGEVIGINTLK--VTA----------GISFAIP
4ri0    LRDSDMDYIQTDAIINYGNAGGPLVNLDGEVIGINTLK--VTA----------GISFAIP
6z05    GLNQYENFIQTDASINPGNSGGALVDSRGYLVGINSAI--LSR--GGG---NNGIGFAIP
2z9i    NQNTVLDAIQTDAAINPGNSGGALVNMNAQLVGVNSAIATLGADSADAQSGSIGLGFAIP
5zvj    DTDTVIDAIQTDASINHGNAGGPLIDMDAQVIGINTAG--KSLSDSAS-----GLGFAIP
3gdv    NPTGRQNFLQTDASINPGNSGGALVNSLGELMGINTLS--FDKSNDGE--TPEGIGFAIP
7co3    GLNTYEDFIQTDAAINPGNAGGALVDAAGNLIGINTAI--FSK--SGG---SQGIGFAIP
3pv5    NIEGVENFIQTDAAIGGGNSGGALVNAKGELIGINTAI--LSP--YGG---NVGIGFAIP
4ynn    NIEGVENFIQTDAAINPGNAGGALVNAKGELIGINTAI--ISP--YGG---NVGIGFAIP
4a9g    NLEGLENFIQTDASINRGNAGGALLNLNGELIGINTAI--LAP--GGG---SVGIGFAIP
2zle    NAENYENFIQTDAAINRGNSGGALVNLNGELIGINTAI--LAP--DGG---NIGIGFAIP
6jjo    NAENYENFIQTDAAINRGNAGGALVNLNGELIGINTAI--LAP--DGG---NIGIGFAIP
5t69    IPDKRVEFIQTDAAINPGNAGGPLLNARGEVIGINTAI--RAD--------ATGIGFAIP
4ic5    NGKSISEAIQTDADINSGNAGGPLLDSYGHTIGVNTAT--FTRKGSGM---SSGVNFAIP
4ic6    TGVTIGGGIQTDAAINPGNAGGPLLDSKGNLIGINTAI--FTQ--TGT---SAGVGFAIP
3qo6    TGRPIQDVIQTDAAINPGNSGGPLLDSSGTLIGINTAI--YSP--SGA---SSGVGFSIP
                :* ** *. **:**. .:  .  :*:                   .:.: **
```

```
4fln     TTVVSHFLTD------YERNGKYTGYPCLGVLLQKLENPALR-----ECLKVP---TN-E
5ilb     TTVVSHFLTD------YERNGKYTGFPVLGIEWQKMENPDLR-----KSMGME---SHQK
5jyk     TPVIVHFIQD------YEKHDKYTGFPVLGIEWQKMENPDLR-----KSMGME---SHQK
5fht     SDRLREFLHRGEKKNSSSGISGSQ-RRYIGVMMLTL-SPSILAELQLREPSFP---DVQH
3nzi     SDKIKKFLTESHDRQ-AKGKAITK-KKYIGIRMMSL-TSSKAKELKDRHRDFP---DVIS
4ri0     SDRITRFLTEFQ----DKQIKDWK-KRFIGIRMRTI-TPSLVDELKASNPDFP---EVSS
6z05     SNMVKDIAKK------LIEKGKID-RGFLGVTILAL-QGDTK-----KAYKNQ------E
2z9i     VDQAKRIADE------LISTGKAS-HASLGVQVT---NDKDT----------------L
5zvj     VNEMKLVANS------LIKDGKIV-HPTLGISTRSV-SNAIA----------------S
3gdv     FQLATKIMDK------LIRDGRVI-RGYIGIGGREI-APLHA-----QGGGID---QL-Q
7co3     TKLALEVMQS------IIEHGQVI-RGWLGVEVKAL-TPELA-----ESLGLG---ET-A
3pv5     INMVKDVAQQ------IIKFGSIH-RGLMGIFVQHL-TPELA-----QAMGYP---EDFQ
4ynn     INMAKDVAQQ------IIKFGSIH-RGLMGIFVQHL-TPELA-----QSMGYA---EDFQ
4a9g     SNMARTLAQQ------LIDFGEIK-RGLLGIKGTEM-SADIA-----KAFNLD---VQ-R
2zle     SNMVKNLTSQ------MVEYGQVK-RGELGIMGTEL-NSELA-----KAMKVD---AQ-R
6jjo     SNMVKNLTSQ------MVEYGQVK-RGELGIMGTEL-NSELA-----KAMKVD----AQR
5t69     IDQAKAIQNT------LAAGGTVP-HPYIGVQMMNI-TVDQA-QQNNRNPNSPFIIPEVD
4ic5     IDTVVRTVPY------LIVYGTAY-RDRLSS------VDKLA-----------------
4ic6     SSTVLKIVPQ------LIQFSKVL-RAGINIELA---PDPVA-----NQLNVR------N
3qo6     VDTVGGIVDQ------LVRFGKVT-RPILGIKF----APDQS----VEQLGV-------S
                                          :.

4fln     GVLVRRVEPTSDASKV-LKE-----------GDVIVSFDDLHVGCEGTVPFRSSERIAFR
5ilb     GVRIRRIEPTAPESQV-LKP-----------SDIILSFDGVNIANDGTVPFRHGERIGFS
5jyk     GVRIRRIEPTAPESQV-LKP-----------SDIILSFDGVNIANDGTVPFRHGERIGFS
5fht     GVLIHKVILGSPAHRAGLRP-----------GDVILAIGEQMV----------QNAEDVY
3nzi     GAYIIEVIPDTPAEAGGLKE-----------NDVIISINGQSV----------VSANDVS
4ri0     GIYVQEVAPNSPSQRGGIQD-----------GDIIVKVNGRPL----------VDSSELQ
6z05     GALITDVQKGSSADEAGLKR-----------GDLVTKVNNKVI----------KSPIDLK
2z9i     GAKIVEVVAGGAAANAGVPK-----------GVVVTKVDDRPI----------NSADALV
5zvj     GAQVANVKAGSPAQKGGILE-----------NDVIVKVGNRAV----------ADSDEFV
3gdv     GIVVNEVSPDGPAANAGIQV-----------NDLIISVDNKPA----------ISALETM
7co3     GIVVAGVYRDGPAARGGLLP-----------GDVILTIDKQEA----------SDGRRSM
3pv5     GALVSQVNPNSPAELAGLKA-----------GDIITQINDTKI----------TQATQVK
4ynn     GALVSQVNQNSPAQLAGLKS-----------GDVIVQINDTKI----------TQATQVK
4a9g     GAFVSEVLPGSGSAKAGVKA-----------GDIITSLNGKPL----------NSFAELR
2zle     GAFVSQVLPNSSAAKAGIKA-----------GDVITSLNGKPI----------SSFAALR
6jjo     GAFVSQVLPNSSAAKAGIKA-----------GDVITSLNGKPI----------SSFAALR
5t69     GILVMRVLPGTPAERAGIRR-----------GDVIVAVDGTPI----------SDGARLQ
4ic5     ------------------------------------------------------------
4ic6     GALVLQVPGKSLAEKAGLHPTSRGFAGNIVLGDIIVAVDDKPV----------KNKAELM
3qo6     GVLVLDAPPSGPAGKAGLQSTKRDGYGRLVLGDIITSVNGTKV----------SNGSDLY
```

```
4fln      YLISQKFAGDIAEIGII-RAGEHKKVQVVLRPRVHLVPYHIDGGQPSYIIVAGLVFTPLS
5ilb      YLISQKYTGDSALVKVL-RNKEILEFNIKLAIHKRLIPAHISGKPPSYFIVAGFVFTTVS
5jyk      YLISQKYTGDSALVKVL-RNKEILEFNIKLAIHKRLIPAHISGKPPSYFIVAGFVFTTVS
5fht      EAVRT---QSQLAVQIR-RGRETLTLYVTPEVTE------LEHHHHHH------------
3nzi      DVIKR---ESTLNMVVR-RGNEDIMITVIPEEID---PRSLEHHHHHH------------
4ri0      EAVLT---ESPLLLEVR-RGNDDLLFSIAPEVVMG------GHHHHHH------------
6z05      NYIGTLEIGQKISLSYE-RDGENKQASFILKGEKENPKGVQS--DLIDGLSLRNLDPRLK
2z9i      AAVRSKAPGATVALTFQDPSGGSRTVQVTLGKAEQ-----LEHHHHHH------------
5zvj      VAVRQLAIGQDAPIEVV-REGRHVTLTVKPDPDSTKLAAALEHHHHHH------------
3gdv      AQVAEIRPGSVIPVVVM-RDDKQLTLQVTIQEYPATN-----------------------
7co3      NQVARTRPGQKISIVVL-RNGQKVNLTAEVGLRPPPAPAPQQKQDGGE------------
3pv5      TTISLLRVGSTVKIIVE-RDNKPLTLSAVVTDIKSHEQKLQSNNPFLYGLALRAFEQESP
4ynn      TTISLLRAGSTAKIKIL-RDNKPLTLDVEVTDIKKHEQKLQSNNPFLYGLALRNFEQESP
4a9g      SRIATTEPGTKVKLGLL-RNGKPLEVEVTLDTSTSSSASAEMITPALEGATLSDGQL---
2zle      AQVGTMPVGSKLTLGLL-RDGKQVNVNLELQQSSQNQVDSSSIFNGIEGAEMSN------
6jjo      AQVGTMPVGSKLTLGLL-RDGKQVNVNLELQQSSQNQVDSSSIFNGIEGAEMSN------
5t69      RIVEQAGLNKALKLDLL-RGDRRLSLTVQTAQLRNPTS----------------------
4ic5      --------------------------------------AALEHHHHHH------------
4ic6      KILDEYSVGDKVTLKIK-RGNEDLELKISLEEKSS-----LEHHHHHH------------
3qo6      RILDQCKVGDEVTVEVL-RGDHKEKISVTLEPKPDESAAALEHHHHHH------------

4fln      EPLIE----EECEDTIGLKLLTKARYSVARFRGEQIVILSQVLANEVNIGYEDMNNQQVL
5ilb      VPYLRSEYGKEYEFDAPVKLLEKHLHAMAQSVDEQLVVVSQVLVSDINIGYEEIVNTQVV
5jyk      VPYLRSEYGKEYEFDAPVKLLEKHLHAMAQSVDEQLVVVSQVLVSDINIGYEEIVNTQVV
5fht      ------------------------------------------------------------
3nzi      ------------------------------------------------------------
4ri0      ------------------------------------------------------------
6z05      DRLQI-------------------------PKDVNGVLVDSVKEKSKGKNSGFQEGDIII
2z9i      ------------------------------------------------------------
5zvj      ------------------------------------------------------------
3gdv      ------------------------------------------------------------
7co3      ------------------------------------------------------------
3pv5      P-----------------------------HGNVIGVQVVGASENSAGWRAGIRPGDIII
4ynn      P-----------------------------HGNVVGVQVVGASETSAGWRAGLRPGDIII
4a9g      ------------------------------KDGGKGIKIDEVVKGSPAAQAGLQKDDVII
2zle      ------------------------------KGKDQGVVVNNVKTGTPAAQIGLKKGDVII
6jjo      ------------------------------KGKDQGVVVNNVKTGTPAAQIGLKKGDVII
5t69      ------------------------------------------------------------
4ic5      ------------------------------------------------------------
4ic6      ------------------------------------------------------------
3qo6      ------------------------------------------------------------
```

```
4fln            KFNGIPIRNIHHLAHLIDMCKDKYLVFEFEDNYVAVLEREASNSASLCILKDYGIPSERS
5ilb            AFNGKPVKNLKGLAGMVENCEDEYMKFNLDYDQIVVLDTKTAKEATLDILTTHCIPSAMS
5jyk            AFNGKPVKNLKGLAGMVENCEDEYMKFNLDYDQIVVLDTKTAKEATLDILTTHCIPSAMS
5fht            ------------------------------------------------------------
3nzi            ------------------------------------------------------------
4ri0            ------------------------------------------------------------
6z05            GVGQSEIKNLKDLEQALKQVNKKEFTKVWVYRNGFATLLVLK------------------
2z9i            ------------------------------------------------------------
5zvj            ------------------------------------------------------------
3gdv            ------------------------------------------------------------
7co3            ------------------------------------------------------------
3pv5            SANKKPVTDVKSLQTIAQEKKKELLVQVLRGPGSMYLLVI--------------------
4ynn            SANKTPVKDIKSLQAVAHEKGKQLLVQVLRGAGALYLLII--------------------
4a9g            GVNRDRVNSIAEMRKVLAAKPAIIALQIVRGNESIYLLMRLEHHHHHH------------
2zle            GANQQAVKNIAELRKVLDSKPSVLALNIQRGDSTIYLLMQ--------------------
6jjo            GANQQAVKNIAELRKVLDSKPSVLALNIQRGDSTIYLLMQ--------------------
5t69            ------------------------------------------------------------
4ic5            ------------------------------------------------------------
4ic6            ------------------------------------------------------------
3qo6            ------------------------------------------------------------

4fln            ADLLEPYVDPIDDTQALDQGIGDSPVSNLEIGFDGLVWA
5ilb            DDLKTEERN------------------------------
5jyk            DDLKTEERN------------------------------
5fht            ---------------------------------------
3nzi            ---------------------------------------
4ri0            ---------------------------------------
6z05            ---------------------------------------
2z9i            ---------------------------------------
5zvj            ---------------------------------------
3gdv            ---------------------------------------
7co3            ---------------------------------------
3pv5            ---------------------------------------
4ynn            ---------------------------------------
4a9g            ---------------------------------------
2zle            ---------------------------------------
6jjo            ---------------------------------------
5t69            ---------------------------------------
4ic5            ---------------------------------------
4ic6            ---------------------------------------
3qo6            ---------------------------------------
```

<u>Figure S2:</u> Frequency of HtrA Repeats in Random Protein Sequences

Comparison of the scoring of random protein sequences to the HtrA repeat sequence. All the randomly generate possible sequences were scored according to a Bernoulli statistical model and the scores were binned. The fraction of sequences that occurred in each bin is plotted against the minimum score for that bin. Note that both axes are log based. A vertical red line indicates the score cuttoff used to identify the HtrA repeat sequences within the sequence unique PDB set.

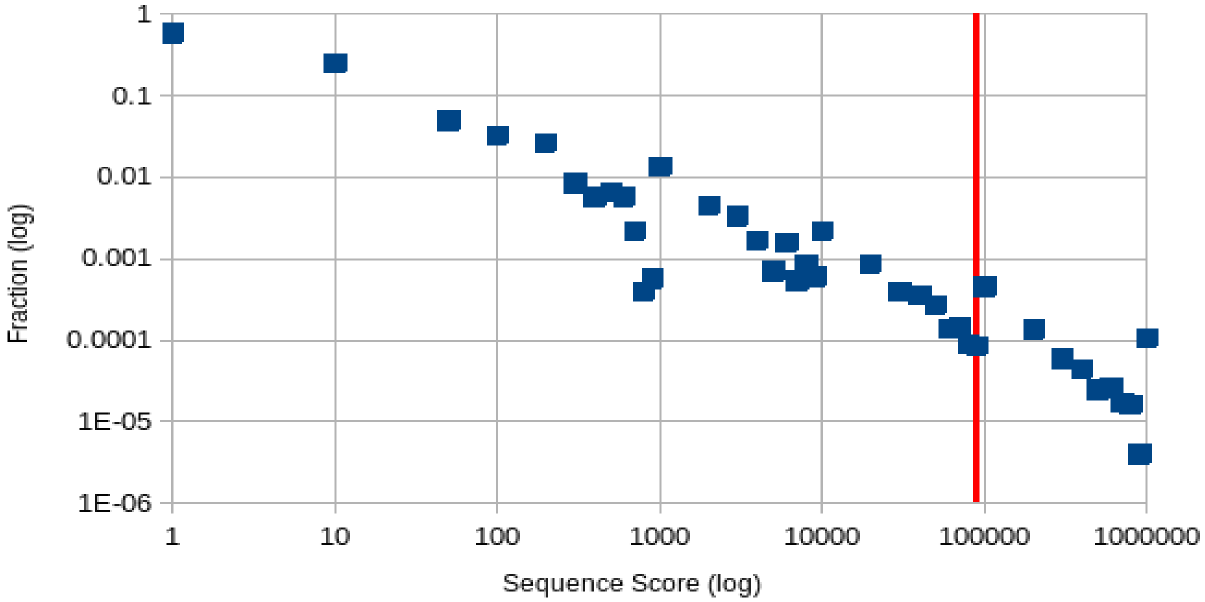

<u>Figure S3:</u> Expected Protein Length of a Random Sequence Matching that Score to Occur

The expected length of a protein in which a sequence scoring X or higher should occur randomly, as extrapolated from randomly generated protein sequences. That is, a protein of length N would be expected to have a section of sequence that scores at least as high as X somewhere within its length. Note the log scale of the y-axis. A vertical red line indicates the scoe cutoff used to identify HtrA repeat seqeunces, the same score as used in Fig. S2. The intersection of this red line and the data points indicates the expected lengt of a protein sequence needed for a random occurrence of a sequence scoring at least that high to occur within that protein, in this case 1031 residues.

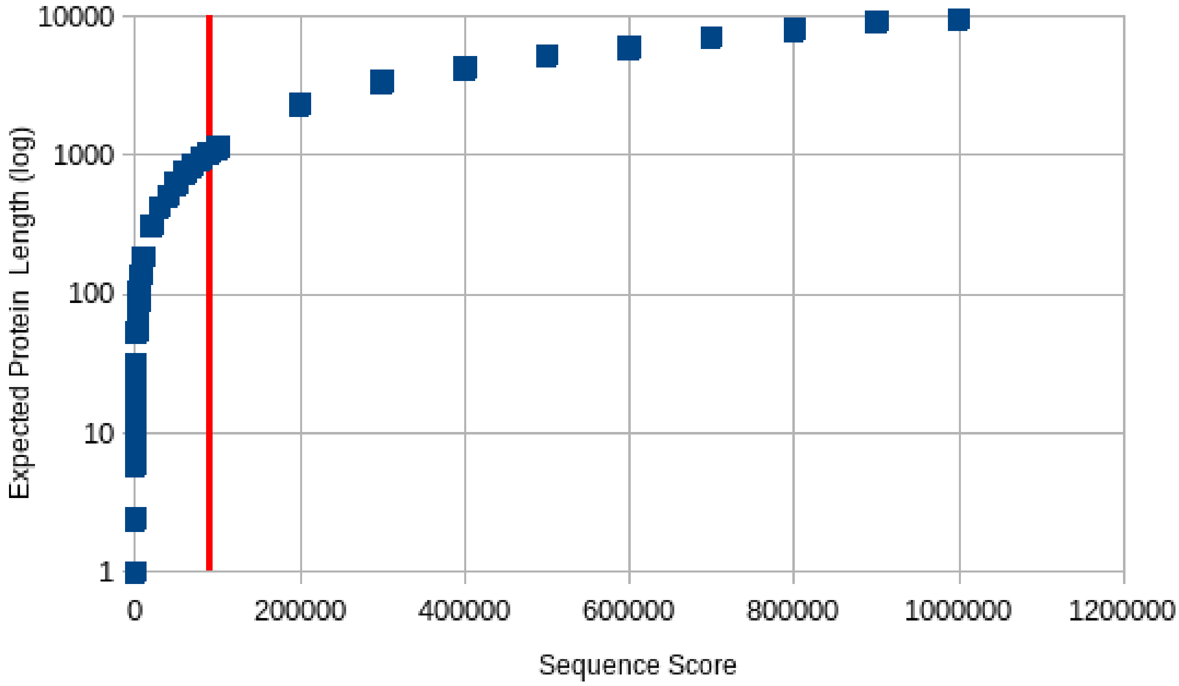

Figure S4: **Identification of the β-barrel Structures in the HtrA Proteases**

The conserved β-barrel structures are explicitly identified by highlighting. The N-terminal protease module in cyan, the C-terminal protease module in magenta, PDZ-like 1 and PDZ-like 2 in orange and violet respectively.

```
CLUSTAL multiple sequence alignment by MUSCLE (3.8)
4fln            --------------------------NAESSNPPQKMAFKAFG-----------------
5ilb            --------------------------GHDASF----------------------------
5jyk            -------------MGSSHH-----HHHHSSGLVPRGSHMASMTGGQQMGRGSEFELLNNE
5fht            ------------------------------MAVP--------------------------
3nzi            -------------MG---------------------------------------------
4ri0            ------------------------------------------------------------
6z05            ------------------------------------------------------------
2z9i            -----------------------------ANMPP--------------------------
5zvj            ------------------------MVDAFTTSKVT-------------------------
3gdv            -------------MRGSHH-----HHHHGRSLNPLS------------------------
7co3            MPKALRFLGWPVLVGVLLALLIIQHNPELVGLPRQEVHVEQ-------------------
3pv5            -------------MRGSHH-----HHHHGSAEPPN-------------------------
4ynn            -------------MRGSHH-----HHHHGSAQDLT-------------------------
4a9g            -----------------------SIPGQVADQAP--------------------------
2zle            ------------------------------------------------------------
6jjo            -------------MGSSHH-----HHHHSSGLVPRGSHMA--------------------
5t69            ---------------------GIDPFTMADDLPPAPVI----------------------
4ic5            -------------MGSSHH-----HHHHSSGLVPRGSHMASAL-----------------
4ic6            -------------MGLGDP-----SVATVEDVSPT-------------------------
3qo6            -------------------------MAAFVVSTPK-------------------------


4fln            -----------SPKKEKKESLSDFSRDQQTDPAKIHDASFLNAVVKVYC-----------
5ilb            ----------------------------------------LNAVVKVYC-----------
5jyk            SEAGNQRTSSPERSRSRLHHSDTKNGDCSNGMIVSTTTESIPAAPSWETVVKVVPSMDAV
5fht            --------------SPPPASPRSQYNFIADVVEKT-----APAVVYIEI-----------
3nzi            --------------QEDPNSLRHKYNFIADVVEKI-----APAVVHIEL-----------
4ri0            --------------LHQLSSPRYKFNFIADVVEKI-----APAVVHIEL-----------
6z05            --------------VLSYHDS----------IKDA-----KKSVVNISTSKTITRANRPS
2z9i            -------------------------GSVEQVAAKV-----VPSVVMLET-----------
5zvj            --------------LSTTGNAQEPAGRFTKVAAAV-----ADSVVTIES-----------
3gdv            --------------TPQFDSTDETPASYNLAVRRA-----APAVVNVYN-----------
7co3            --------------APLLSRLQEGPVSYANAVSRA-----APAVANLYTTKMVSKPSHPL
3pv5            --------------MPSMAPV----------LKNI-----MPAIVNVAVQGYLP------
4ynn            -----------------------NMPSLAPVLKNA-----MPAIVNVAVQGYLPNNM---
4a9g            --------------LPSLAPM----------LEKV-----LPAVVSVRVEGTASQGQK--
2zle            -------------AETSSATTAQQMPSLAPMLEKV-----MPSVVSINVEGSTTVNTPR-
6jjo            --------------ETSSATTAQQMPSLAPMLEKV-----MPSVVSINVEGSTTVNTPR-
5t69            --------------TAQASVPLTSESFVAAAVSRS-----GPAVVRIDTETVVTRRTDPI
4ic5            -----------EQFKEKEEELEEEEERNVNLFQKT-----SPSVVYIEA-----------
4ic6            --------------VFPAGPLFPTEGRIVQLFEKN-----TYSVVNIFD-----------
3qo6            --------------KLQTDEL-----ATVRLFQEN-----TPSVVYITN-----------
                                                          :
```

```
4fln        ----------THTAPDYSLPWQKQ----------------------RQFTSTGSAFMIG-
5ilb        ----------THTAPDYSLPWQKQ----------------------RQFTSTGSAFMIG-
5jyk        V-----KVFCVHTEPNFSLPWQRK----------------------RQYSSGSSGFIIG-
5fht        --------LDRHPFLGREVPI-----------------------------SNGSGFVVAA
3nzi        --------FRKLPFSKREVPV-----------------------------ASGSGFIVSE
4ri0        --------FLRHPLFGRNVPL-----------------------------SSGSGFIMSE
6z05        PLDDFFNDPYFKQFFDFDFPQRKGKNDK------------------EVVSSLGSGVIISK
2z9i        -------------DLGRQSEE-------------------------------GSGIILSA
5zvj        --------------VSDQEGM------------------------------QGSGVIVDG
3gdv        --------RGLNTNSHNQLEIR----------------------------TLGSGVIMDQ
7co3        F-----DDPMFRRFFGDNLPQQK-----------------------RMESSLGSAVIMSA
3pv5        ------NDVTPPGSAGNDEENQPNNRPPQSRMPEKG----------RKFESIGSGVIIDP
4ynn        ------ASGNADDDDGENSKQPSRIPEKG-----------------RKFESIGSGVIIDP
4a9g        ------IPEEFKKFFGDDLPDQPA----------------------QPFEGLGSGVIINA
2zle        ------MPRNFQQFFGDDSPFCQEGSPFQSSPFCQGGQGGNGGGQQQKFMALGSGVIIDA
6jjo        ------MPRNFQQFFGDDSPFCQEGSPFQSSPFCQGGQGGNGGGQQQKFMALGSGVIIDA
5t69        L-----DDPFFQEFFGRSFPVPPRE---------------------RRIAGQGSGFIIDN
4ic5        ------IEL-PKTSSGDILTDEEN----------------------GKIEGTGSGFVWDK
4ic6        ------VTLRPQLKMTGVVEIP---------------------------EGNGSGVVWDG
3qo6        ------LAVRQDAFTLDVLEVP---------------------------QGSGSGFVWDK
                                                                .*..:

4fln        -DGKLLTNAHCVEHD--T---------QVKVKRRGDDR-KYVAKVLVRGVDCDIALLSVE
5ilb        -DGKLLTNAHCVEHD--T---------QVKVKRRGDDR-KYVAKVLVRGVDCDIALLSVE
5jyk        -GRRVLTNAHSVEHH--T---------QVKLKKRGSDT-KYLATVLAIGTECDIALLTVT
5fht        -DGLIVTNAHVVADR--R---------RVRVRLLSG-D-TYEAVVTAVDPKADIATLRIQ
3nzi        -DGLIVTNAHVVTNK--H---------RVKVELKNG-A-TYEAKIKDVDEKADIALIKID
4ri0        -AGLIITNAHVVSSN--SAAPGRQ---QLKVQLQNG-D-SYEATIKDIDKKSDIATIKIH
6z05        -DGYIVTNNHVVDDA--D---------TITVNLPGSDI-EYKAKLIGKDPKTDLAVIKIE
2z9i        -EGLILTNNHVIAAA--AKPPLGSPPPKTTVTFSDG-R-TAPFTVVGADPTSDIAVVRVQ
5zvj        -RGYIVTNNHVISEA--ANNPSQF---KTTVVFNDG-K-EVPANLVGRDPKTDLAVLKVD
3gdv        -RGYIITNKHVINDA--D---------QIIVALQDG-R-VFEALLVGSDSLTDLAVLKIN
7co3        -EGYLLTNNHVTAGA--D---------QIIVALRDG-R-ETIAQLVGSDPETDLAVLKID
3pv5        NNGVIITNDHVIRNA--S---------LITVTLQDG-R-RLKARLIGGDSETDLAVLKID
4ynn        KNGIIITNDHVIRNA--N---------LITVTLQDG-R-RLKARLIGGDSETDLAVLKID
4a9g        SKGYVLTNNHVINQA--Q---------KISIQLNDG-R-EFDAKLIGSDDQSDIALLQIQ
2zle        DKGYVVTNNHVVDNA--T---------VIKVQLSDG-R-KFDAKMVGKDPRSDIALIQIQ
6jjo        DKGYVVTNNHVVDNA--T---------VIKVQLSDG-R-KFDAKMVGKDPRSDIALIQIQ
5t69        -SGIILTNAHVVDGA--S---------KVVVTLRDG-R-TFDGQVRGTDEVTDLAVVKIE
4ic5        -LGHIVTNYHVIAKL--ATDQFGLQRCKVSLVDAKGTRFSKEGKIVGLDPDNDLAVLKIE
4ic6        -QGYIVTNYHVIGNALSRNPSPGDVVGRVNILASDGVQKNFEGKLVGADRAKDLAVLKVD
3qo6        -QGHIVTNYHVIRGA--S---------DLRVTLADQ-T-TFDAKVVGFDQDKDVAVLRID
                ::** *                    :             .   *:* : :

4fln        SEDFWKGAEPLRLGHLPRLQDSVTVVGYPLGG----DTISVTKGVVSRIEVT-----SYA
5ilb        SEDFWKGAEPLRLGHLPRLQDSVTVVGYPLGG----DTISVTKGVVSRIEVT-----SYA
5jyk        DDEFWEGVSPVEFGDLPALQDAVTVVGYPIGG----DTISVTSGVVSRMEIL-----SYV
5fht        TKEP-LPTLPLGRSADVRQGEFVVAMGSPFAL-----QNTITSGIVSSAQRPAR---DLG
3nzi        HQGK-LPVLLLGRSSELRPGEFVVAIGSPFSL-----QNTVTTGIVSTTQRGGK---ELG
4ri0        PKKK-LPVLLLGHSADLRPGEFVVAIGSPFAL-----QNTVTTGIVSTAQREGR---ELG
6z05        ANN--LSAITFTNSDDLMEGDVVFALGNPFGV-----GFSVTSGIISALNK------DNI
2z9i        GVSG-LTPISLGSSSDLRVGQPVLAIGSPLGL-----EGTVTTGIVSALNRPVSTTGEAG
5zvj        NVDN-LTVARLGDSSKVRVGDEVLAVGAPLGL-----RSTVTQGIVSALHRPVPLSGEGS
3gdv        ATGG-LPTIPINARRVPHIGDVVLAIGNPYNL-----GQTITQGIISATGR------IGL
7co3        LKN--LPAMTLGRSDGIRTGDVCLAIGNPFGV-----GQTVTMGIISATGR------NQL
3pv5        AKN--LKSLVIGDSDKLEVGDFVVAIGNPFGLNSFGNSQSATFGIVSALKR------SDL
4ynn        AKN--LKSLVIGDSDKLEVGDYVVAIGNPFGLNSFGNSQSATFGIVSALKR------SDL
4a9g        NPSK-LTQIAIADSDKLRVGDFAVAVGNPFGL-----GQTATSGIVSALGR------SGL
2zle        NPKN-LTAIKMADSDALRVGDYTVAIGNPFGL-----GETVTSGIVSALGR------SGL
6jjo        NPKN-LTAIKMADSDALRVGDYTVAIGNPFGL-----GETVTSGIVSALGR------SGL
5t69        PQGSALPVAPLGTSSNLQVGDWAIAVGNPVGL-----DNTVTLGIISTLGRSAA---QAG
4ic5        TEGRELNPVVLGTSNDLRVGQSCFAIGNPYGY-----ENTLTIGVVSGLGREI----PSP
4ic6        APETLLKPIKVGQSNSLKVGQQCLAIGNPFGF-----DHTLTVGVISGLNRDI----FSQ
3qo6        APKNKLRPIPVGVSADLLVGQKVFAIGNPFGL-----DHTLTTGVISGLRREIS---SAA
                      .         :   .:* *          : * *::*
```

```
4fln      HGSSDLLGIQIDAAINPGNSGGPAFNDQGECIGVAFQV--YRSEETE------NIGYVIP
5ilb      HGSSDLLGIQIDAAINPGNSGGPAFNDQGECIGVAFQV--YRSEETE------NIGYVIP
5jyk      HGSTELLGLQIDAAINSGNSGGPAFNDKGKCVGIAFQS--LKHEDAE------NIGYVIP
5fht      LPQTNVEYIQTDAAIDFGNAGGPLVNLDGEVIGVNTMK--VTA----------GISFAIP
3nzi      LRNSDMDYIQTDAIINYGNSGGPLVNLDGEVIGINTLK--VTA----------GISFAIP
4ri0      LRDSDMDYIQTDAIINYGNAGGPLVNLDGEVIGINTLK--VTA----------GISFAIP
6z05      GLNQYENFIQTDASINPGNSGGALVDSRGYLVGINSAI--LSR--GGG---NNGIGFAIP
2z9i      NQNTVLDAIQTDAAINPGNSGGALVNMNAQLVGVNSAIATLGADSADAQSGSIGLGFAIP
5zvj      DTDTVIDAIQTDASINHGNAGGPLIDMDAQVIGINTAG--KSLSDSAS-----GLGFAIP
3gdv      NPTGRQNFLQTDASINPGNSGGALVNSLGELMGINTLS--FDKSNDGE--TPEGIGFAIP
7co3      GLNTYEDFIQTDAAINPGNAGGALVDAAGNLIGINTAI--FSK--SGG---SQGIGFAIP
3pv5      NIEGVENFIQTDAAIGGGNSGGALVNAKGELIGINTAI--LSP--YGG---NVGIGFAIP
4ynn      NIEGVENFIQTDAAINPGNAGGALVNAKGELIGINTAI--ISP--YGG---NVGIGFAIP
4a9g      NLEGLENFIQTDASINRGNAGGALLNLNGELIGINTAI--LAP--GGG---SVGIGFAIP
2zle      NAENYENFIQTDAAINRGNSGGALVNLNGELIGINTAI--LAP--DGG---NIGIGFAIP
6jjo      NAENYENFIQTDAAINRGNAGGALVNLNGELIGINTAI--LAP--DGG---NIGIGFAIP
5t69      IPDKRVEFIQTDAAINPGNAGGPLLNARGEVIGINTAI--RAD--------ATGIGFAIP
4ic5      NGKSISEAIQTDADINSGNAGGPLLDSYGHTIGVNTAT--FTRKGSGM---SSGVNFAIP
4ic6      TGVTIGGGIQTDAAINPGNAGGPLLDSKGNLIGINTAI--FTQ--TGT---SAGVGFAIP
3qo6      TGRPIQDVIQTDAAINPGNSGGPLLDSSGTLIGINTAI--YSP--SGA---SSGVGFSIP
                  :* ** *. **:**. .:  .  :*:                   .:.: **

4fln      TTVVSHFLTD------YERNGKYTGYPCLGVLLQKLENPALR-----ECLKVP---TN-E
5ilb      TTVVSHFLTD------YERNGKYTGFPVLGIEWQKMENPDLR-----KSMGME---SHQK
5jyk      TPVIVHFIQD------YEKHDKYTGFPVLGIEWQKMENPDLR-----KSMGME---SHQK
5fht      SDRLREFLHRGEKKNSSSGISGSQ-RRYIGVMMLTL-SPSILAELQLREPSFP---DVQH
3nzi      SDKIKKFLTESHDRQ-AKGKAITK-KKYIGIRMMSL-TSSKAKELKDRHRDFP---DVIS
4ri0      SDRITRFLTEFQ----DKQIKDWK-KRFIGIRMRTI-TPSLVDELKASNPDFP---EVSS
6z05      SNMVKDIAKK------LIEKGKID-RGFLGVTILAL-QGDTK-----KAYKNQ------E
2z9i      VDQAKRIADE------LISTGKAS-HASLGVQVT---NDKDT-----------------L
5zvj      VNEMKLVANS------LIKDGKIV-HPTLGISTRSV-SNAIA-----------------S
3gdv      FQLATKIMDK------LIRDGRVI-RGYIGIGGREI-APLHA-----QGGGID---QL-Q
7co3      TKLALEVMQS------IIEHGQVI-RGWLGVEVKAL-TPELA-----ESLGLG---ET-A
3pv5      INMVKDVAQQ------IIKFGSIH-RGLMGIFVQHL-TPELA-----QAMGYP---EDFQ
4ynn      INMAKDVAQQ------IIKFGSIH-RGLMGIFVQHL-TPELA-----QSMGYA---EDFQ
4a9g      SNMARTLAQQ------LIDFGEIK-RGLLGIKGTEM-SADIA-----KAFNLD---VQ-R
2zle      SNMVKNLTSQ------MVEYGQVK-RGELGIMGTEL-NSELA-----KAMKVD---AQ-R
6jjo      SNMVKNLTSQ------MVEYGQVK-RGELGIMGTEL-NSELA-----KAMKVD----AQR
5t69      IDQAKAIQNT------LAAGGTVP-HPYIGVQMMNI-TVDQA-QQNNRNPNSPFIIPEVD
4ic5      IDTVVRTVPY------LIVYGTAY-RDRLSS------VDKLA------------------
4ic6      SSTVLKIVPQ------LIQFSKVL-RAGINIELA---PDPVA-----NQLNVR------N
3qo6      VDTVGGIVDQ------LVRFGKVT-RPILGIKF----APDQS----VEQLGV-------S
                                      :.

4fln      GVLVRRVEPTSDASKV-LKE-----------GDVIVSFDDLHVGCEGTVPFRSSERIAFR
5ilb      GVRIRRIEPTAPESQV-LKP-----------SDIILSFDGVNIANDGTVPFRHGERIGFS
5jyk      GVRIRRIEPTAPESQV-LKP-----------SDIILSFDGVNIANDGTVPFRHGERIGFS
5fht      GVLIHKVILGSPAHRAGLRP-----------GDVILAIGEQMV----------QNAEDVY
3nzi      GAYIIEVIPDTPAEAGGLKE-----------NDVIISINGQSV----------VSANDVS
4ri0      GIYVQEVAPNSPSQRGGIQD-----------GDIIVKVNGRPL----------VDSSELQ
6z05      GALITDVQKGSSADEAGLKR-----------GDLVTKVNNKVI----------KSPIDLK
2z9i      GAKIVEVVAGGAAANAGVPK-----------GVVVTKVDDRPI----------NSADALV
5zvj      GAQVANVKAGSPAQKGGILE-----------NDVIVKVGNRAV----------ADSDEFV
3gdv      GIVVNEVSPDGPAANAGIQV-----------NDLIISVDNKPA----------ISALETM
7co3      GIVVAGVYRDGPAARGGLLP-----------GDVILTIDKQEA----------SDGRRSM
3pv5      GALVSQVNPNSPAELAGLKA-----------GDIITQINDTKI----------TQATQVK
4ynn      GALVSQVNQNSPAQLAGLKS-----------GDVIVQINDTKI----------TQATQVK
4a9g      GAFVSEVLPGSGSAKAGVKA-----------GDIITSLNGKPL----------NSFAELR
2zle      GAFVSQVLPNSSAAKAGIKA-----------GDVITSLNGKPI----------SSFAALR
6jjo      GAFVSQVLPNSSAAKAGIKA-----------GDVITSLNGKPI----------SSFAALR
5t69      GILVMRVLPGTPAERAGIRR-----------GDVIVAVDGTPI----------SDGARLQ
4ic5      ------------------------------------------------------------
4ic6      GALVLQVPGKSLAEKAGLHPTSRGFAGNIVLGDIIVAVDDKPV----------KNKAELM
3qo6      GVLVLDAPPSGPAGKAGLQSTKRDGYGRLVLGDIITSVNGTKV----------SNGSDLY
```

```
4fln                YLISQKFAGDIAEIGII-RAGEHKKVQVVLRPRVHLVPYHIDGGQPSYIIVAGLVFTPLS
5ilb                YLISQKYTGDSALVKVL-RNKEILEFNIKLAIHKRLIPAHISGKPPSYFIVAGFVFTTVS
5jyk                YLISQKYTGDSALVKVL-RNKEILEFNIKLAIHKRLIPAHISGKPPSYFIVAGFVFTTVS
5fht                EAVRT---QSQLAVQIR-RGRETLTLYVTPEVTE------LEHHHHHH------------
3nzi                DVIKR---ESTLNMVVR-RGNEDIMITVIPEEID---PRSLEHHHHHH------------
4ri0                EAVLT---ESPLLLEVR-RGNDDLLFSIAPEVVMG------GHHHHHH------------
6z05                NYIGTLEIGQKISLSYE-RDGENKQASFILKGEKENPKGVQS--DLIDGLSLRNLDPRLK
2z9i                AAVRSKAPGATVALTFQDPSGGSRTVQVTLGKAEQ-----LEHHHHHH------------
5zvj                VAVRQLAIGQDAPIEVV-REGRHVTLTVKPDPDSTKLAAALEHHHHHH------------
3gdv                AQVAEIRPGSVIPVVVM-RDDKQLTLQVTIQEYPATN-----------------------
7co3                NQVARTRPGQKISIVVL-RNGQKVNLTAEVGLRPPPAPAPQQKQDGGE------------
3pv5                TTISLLRVGSTVKIIVE-RDNKPLTLSAVVTDIKSHEQKLQSNNPFLYGLALRAFEQESP
4ynn                TTISLLRAGSTAKIKIL-RDNKPLTLDVEVTDIKKHEQKLQSNNPFLYGLALRNFEQESP
4a9g                SRIATTEPGTKVKLGLL-RNGKPLEVEVTLDTSTSSSASAEMITPALEGATLSDGQL---
2zle                AQVGTMPVGSKLTLGLL-RDGKQVNVNLELQQSSQNQVDSSSIFNGIEGAEMSN------
6jjo                AQVGTMPVGSKLTLGLL-RDGKQVNVNLELQQSSQNQVDSSSIFNGIEGAEMSN------
5t69                RIVEQAGLNKALKLDLL-RGDRRLSLTVQTAQLRNPTS----------------------
4ic5                --------------------------------------AALEHHHHHH------------
4ic6                KILDEYSVGDKVTLKIK-RGNEDLELKISLEEKSS-----LEHHHHHH------------
3qo6                RILDQCKVGDEVTVEVL-RGDHKEKISVTLEPKPDESAAALEHHHHHH------------

4fln                EPLIE----EECEDTIGLKLLTKARYSVARFRGEQIVILSQVLANEVNIGYEDMNNQQVL
5ilb                VPYLRSEYGKEYEFDAPVKLLEKHLHAMAQSVDEQLVVVSQVLVSDINIGYEEIVNTQVV
5jyk                VPYLRSEYGKEYEFDAPVKLLEKHLHAMAQSVDEQLVVVSQVLVSDINIGYEEIVNTQVV
5fht                ------------------------------------------------------------
3nzi                ------------------------------------------------------------
4ri0                ------------------------------------------------------------
6z05                DRLQI-------------------------PKDVNGVLVDSVKEKSKGKNSGFQEGDIII
2z9i                ------------------------------------------------------------
5zvj                ------------------------------------------------------------
3gdv                ------------------------------------------------------------
7co3                ------------------------------------------------------------
3pv5                P-----------------------------HGNVIGVQVVGASENSAGWRAGIRPGDIII
4ynn                P-----------------------------HGNVVGVQVVGASETSAGWRAGLRPGDIII
4a9g                ------------------------------KDGGKGIKIDEVVKGSPAAQAGLQKDDVII
2zle                ------------------------------KGKDQGVVVNNVKTGTPAAQIGLKKGDVII
6jjo                ------------------------------KGKDQGVVVNNVKTGTPAAQIGLKKGDVII
5t69                ------------------------------------------------------------
4ic5                ------------------------------------------------------------
4ic6                ------------------------------------------------------------
3qo6                ------------------------------------------------------------

4fln                KFNGIPIRNIHHLAHLIDMCKDKYLVFEFEDNYVAVLEREASNSASLCILKDYGIPSERS
5ilb                AFNGKPVKNLKGLAGMVENCEDEYMKFNLDYDQIVVLDTKTAKEATLDILTTHCIPSAMS
5jyk                AFNGKPVKNLKGLAGMVENCEDEYMKFNLDYDQIVVLDTKTAKEATLDILTTHCIPSAMS
5fht                ------------------------------------------------------------
3nzi                ------------------------------------------------------------
4ri0                ------------------------------------------------------------
6z05                GVGQSEIKNLKDLEQALKQVNKKEFTKVWVYRNGFATLLVLK------------------
2z9i                ------------------------------------------------------------
5zvj                ------------------------------------------------------------
3gdv                ------------------------------------------------------------
7co3                ------------------------------------------------------------
3pv5                SANKKPVTDVKSLQTIAQEKKKELLVQVLRGPGSMYLLVI--------------------
4ynn                SANKTPVKDIKSLQAVAHEKGKQLLVQVLRGAGALYLLII--------------------
4a9g                GVNRDRVNSIAEMRKVLAAKPAIIALQIVRGNESIYLLMRLEHHHHHH------------
2zle                GANQQAVKNIAELRKVLDSKPSVLALNIQRGDSTIYLLMQ--------------------
6jjo                GANQQAVKNIAELRKVLDSKPSVLALNIQRGDSTIYLLMQ--------------------
5t69                ------------------------------------------------------------
4ic5                ------------------------------------------------------------
4ic6                ------------------------------------------------------------
3qo6                ------------------------------------------------------------
```

```
4fln            ADLLEPYVDPIDDTQALDQGIGDSPVSNLEIGFDGLVWA
5ilb            DDLKTEERN------------------------------
5jyk            DDLKTEERN------------------------------
5fht            ---------------------------------------
3nzi            ---------------------------------------
4ri0            ---------------------------------------
6z05            ---------------------------------------
2z9i            ---------------------------------------
5zvj            ---------------------------------------
3gdv            ---------------------------------------
7co3            ---------------------------------------
3pv5            ---------------------------------------
4ynn            ---------------------------------------
4a9g            ---------------------------------------
2zle            ---------------------------------------
6jjo            ---------------------------------------
5t69            ---------------------------------------
4ic5            ---------------------------------------
4ic6            ---------------------------------------
3qo6            ---------------------------------------
```

Figure S5: Secondary Structures of the HtrA Modules

Cartoon representation of the secondary structures of β-barrel modules in the HtrA proteases. A) Arrangement of the strands in the protease domain modules. Strands are colored from N- to C-terminus in the order orange, yellow, green, cyan, blue, and magenta respectively. B) Arrangement of the strands in the PDZ-like domain modules. The PDZ-like domain modules have four strands that correspond to the same spatial positions in the protease domain modules and those strands are colored their equivalent color. There are no equivalent strands in the orange or magenta positions in the PDZ-like modules. Additionally, the first strand in the sequence which has been rotated out of the barrel is colored in grey. A sixth strand is present in some of the proteins, but it too has also been roated out of the barrel and is colored in grey in the figure as well. Note that the alternating, antiparallel arrangement of the strands is maintained in the PDZ-like domains, even if it is formally reversed (as the definition of positive and negative is an arbitrary choice).

A

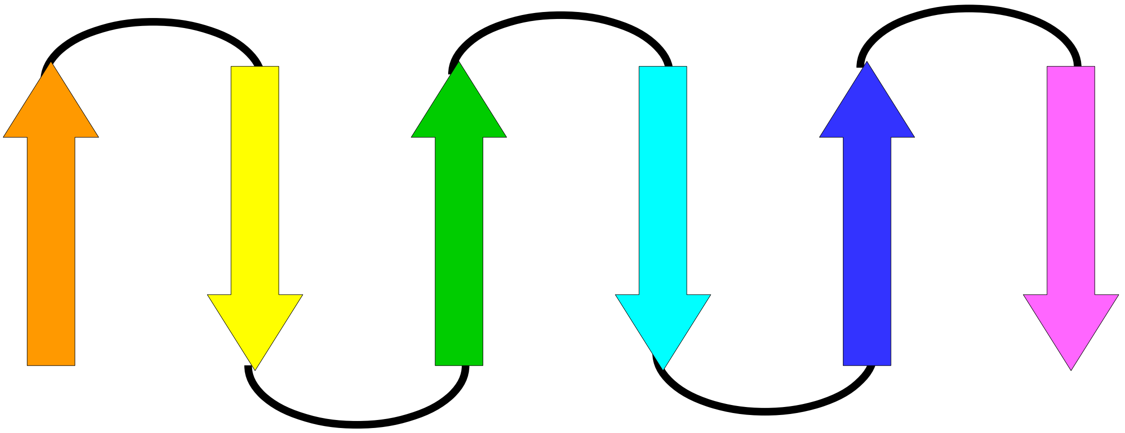

B

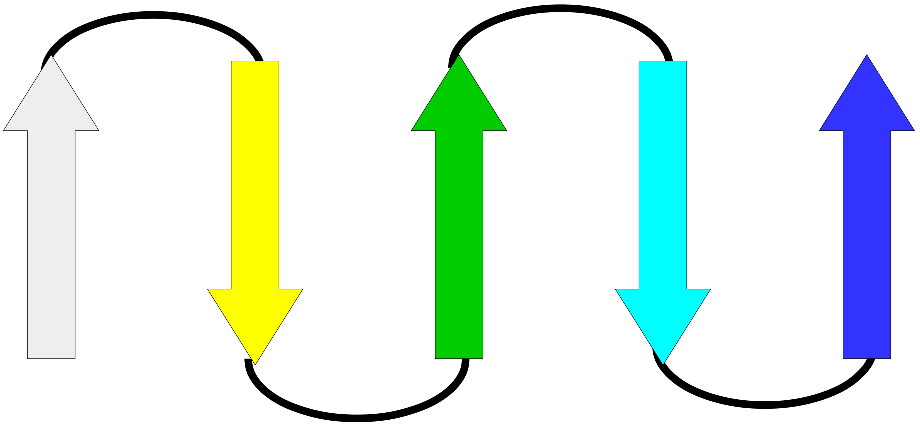

<u>Figure S6:</u> Structures of the HtrA Modules

Cartoon representation of the modules of the sequence unique HtrA protease structures showing the alpha helices (red) and beta strands (yellow). Coil regions are hidden to simplify the images. RMSDs are reported from PyMol using cealign to PDB ID 7co3. Figures were made with PyMol: A) all the N-terminal protease modules (RMSD mean = 1.476 Å, median = 1.438 Å) B) all the C-terminal protease modules (RMSD mean = 2.382 Å, median = 2.397 Å) C) all the PDZ modules (RMSD mean = 2.687 Å, median = 2.598 Å) D) all modules (both protease and PDZ-like) (RMSD mean = 2.237 Å, median = 2.320 Å) E) all three modules from (PDB ID 5t69) with the individual strands colored based on their structural position (colors from N to C in the protease domain: orange, yellow, green, blue, and magenta)(RMSD mean = 1.748 Å, median = 1.816 Å)

A)

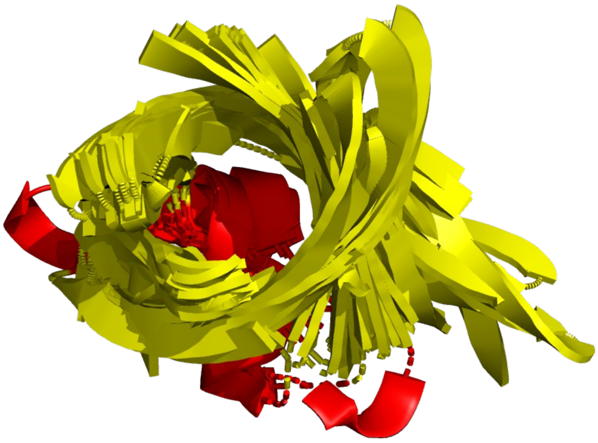

B)

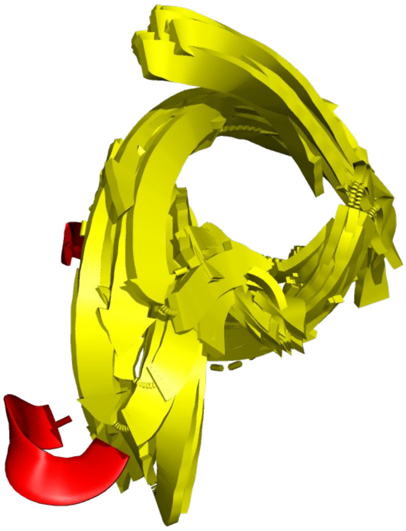

C)

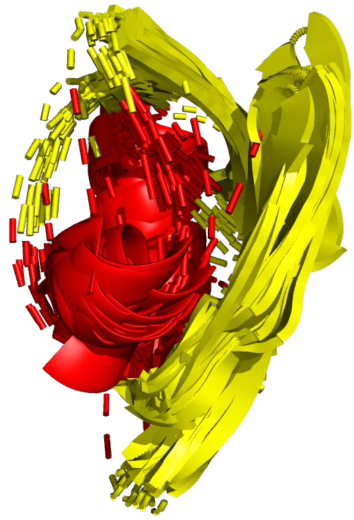

D)

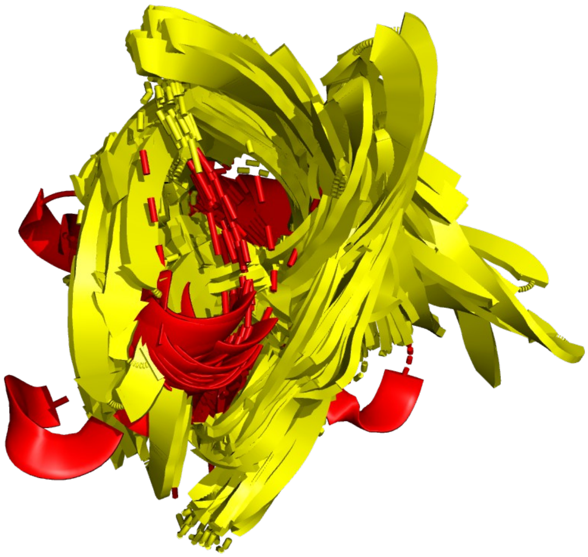

E)

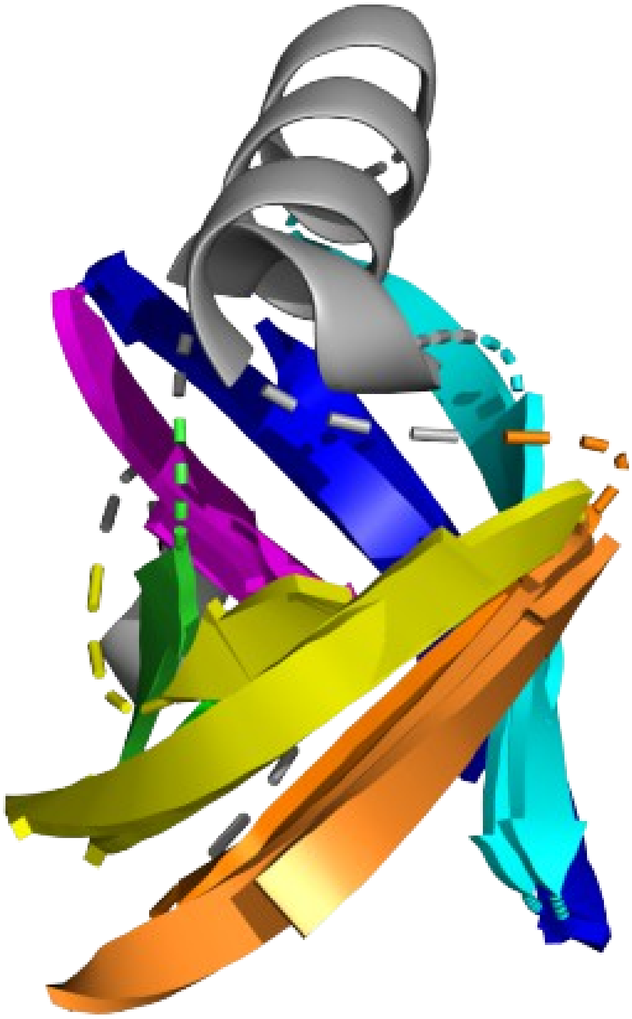

Table S1: Domain Sequence Similarity Comparison

Comparision of the average sequence identity between the different domains in the HtrA protease PDB structure set. Identity was calculated by pairwise comparision using the Needleman-Wunsch alignment method. Domains were defined using the PDB structures.

% ID (mean)	Protease	PDZ-like 1	PDZ-like 2
**Protease**	20.1		
**PDZ-like 1**	18.2	21.2	
**PDZ-like 2**	16.4	19.9	26.1

% ID (median)	Protease	PDZ-like 1	PDZ-like 2
**Protease**	15.4		
**PDZ-like 1**	15.3	15.5	
**PDZ-like 2**	14.2	16.3	17.1

<u>Table S2:</u> Unmodified RMSD Comparison Table

RMSD (Å) calculated (CE-align in PyMol) comparing modules from a given protein structure to its own other modules.

Protein	Prot1-Prot2	Prot1-PDZ1	Prot2-PDZ1	Prot1-PDZ2	Prot2-PDZ2	PDZ1-PDZ2
2zle	4.67	5.39	9.87	9.20	8.58	2.89
2z9i	4.65	7.00	9.95			
3gdv	4.56	10.31	8.33			
3nzi	4.20					
3pv5	3.28	9.09	9.70			
3qo6	3.94	9.47	9.21			
4a9g	4.13	9.67	9.41	7.62	9.79	4.52
4fln	3.56	9.57	9.20	10.26	10.51	4.81
4ic5	5.26					
4ic6	4.43	8.83	9.80			
4ri0	5.02	7.70	6.89			
4ynn	3.40	9.18	10.27	10.34	9.60	2.41
5fht	4.19	9.52	9.58			
5ilb	3.42	8.85	9.16	10.05	7.97	4.95
5jyk	3.09	9.05	9.53	8.32	11.16	5.45
5t69	2.75	10.54	5.85			
5zvj	4.54	5.34	9.87			
6jjo	2.86	9.13	9.48	9.21	7.01	2.18
6z05	6.05	9.36	10.22	8.80	9.93	4.13
7co3	3.20	9.78	9.94			
**Mean =**	4.06	8.77	9.24	9.22	9.32	3.92
**Median =**	4.16	9.16	9.56	9.20	9.70	4.32

Table S3: β-barrel Comparison Summary Table

Summary table for comparisons of the β-barrel modules in the HtrA proteases. "all" indicates that pairwise comparisons of all modules were included to calculate the comparison value. The listing "self" indicates only pairwise comparisons between protease or between PDZ-like modules were included. The listing "other" indicates pairwise comparisons of protease domains to PDZ-like domains were used to calculate the comparison value. The listing "proteases" indicates only comparsions between protease modules were used. "PDZ" indicates that only comparisions between PDZ-like modules were used to calculate the comparison values while "PDZ1" indicates only comparisions to the first PDZ-like domain were used.

CEALIGN to self (RMSD, Å)

	Proteases	Proteases to PDZ1
**Mean =**	4.060	9.002
**Median =**	4.160	9.441

CEALIGN loops removed (RMSD, Å)

	All	Self	Other
**Mean =**	6.444	5.392	7.056
**Median =**	6.789	5.002	7.460

CEALIGN to 5m3n (RMSD, Å)

	N-protease	C-protease	PDZ1
**Mean =**	2.890	2.936	2.124
**Median =**	2.500	2.819	1.677

TM Align (RMSD, Å)

	Proteases	PDZ	Other
**Mean =**	2.804	2.273	3.724
**Median =**	2.780	2.215	3.695

TM Align (TM score)

	Proteases	PDZ	Other
**Mean =**	0.552	0.552	0.298
**Median =**	0.549	0.549	0.299
